# Entre retrocessos e avanços: a trajetória da insegurança alimentar em domicílios negros no Brasil (2013-2023)

**DOI:** 10.1590/0102-311XPT165825

**Published:** 2026-07-31

**Authors:** Gleiciane Bueno da Silva Luiz, Lissandra Amorim Santos Degner, Aline Alves Ferreira, Rosana Salles-Costa

**Affiliations:** 1 Instituto de Nutrição Josué de Castro, Universidade Federal do Rio de Janeiro, Rio de Janeiro, Brasil.; 2 Secretaria de Atenção Primária à Saúde, Ministério da Saúde, Brasília, Brasil.; 3 Secretaria Extraordinária de Combate à Pobreza e à Fome, Ministério do Desenvolvimento e Assistência Social, Família e Combate à Fome, Brasília, Brasil.

**Keywords:** Insegurança Alimentar, Raça, População Negra, Food Insecurity, Race, Black People, Inseguridad Alimentaria, Raza, Población Negra

## Abstract

O objetivo do estudo foi avaliar a trajetória da insegurança alimentar no país segundo as intersecções de sexo e raça/cor ao longo de 2013 a 2023. Microdados de inquéritos populacionais foram avaliados (*Pesquisa Nacional por Amostra de Domicílios*, PNAD 2013; n = 116.196; *Pesquisa de Orçamentos Familiares* 2018; n = 57.920; PNAD Contínua 2023; n = 173.676). Segurança alimentar e insegurança alimentar foram estimadas com base nos perfis da chefia dos domicílios definidos com base no sexo e raça/cor autorreferidos (homem branco, mulher branca, homem pardo, mulher parda, homem preto, mulher preta). Estimou-se as razões de risco relativo (RRR) por modelos de regressão logística multinomial para testar as trajetórias das associações com a insegurança alimentar. Entre 2013 e 2017/2018, a segurança alimentar reduziu em todos os perfis, seguida pelo aumento no período de 2018 a 2023 principalmente entre famílias chefiadas por mulheres pretas (valor de p < 0,001). As formas mais severas da insegurança alimentar permaneceram associadas significativamente entre famílias chefiadas por pessoas pretas ou pardas, sendo maiores naquelas em que a mulher preta ocupava essa posição. Ao longo dos 10 anos dos inquéritos, famílias em insegurança alimentar leve chefiadas por uma mulher branca reduziram os valores de RRR (RRR = 1,2; IC95%: 1,1-1,4). Para aquelas em insegurança alimentar grave, a maior redução de RRR observou-se entre as famílias chefiadas por uma mulher preta (RRR = 2,1; IC95%: 1,8-2,5). Embora a insegurança alimentar tenha reduzido no país em 2023, desigualdades raciais persistem, reiterando a necessidade de políticas públicas para reduzir as iniquidades estruturais enfrentadas pela população negra.

## Abstract (en)

This study aimed to evaluate the trajectory of food insecurity in Brazil according to the intersections of sex and race/skin color from 2013 to 2023. Microdata from population surveys were evaluated (*Brazilian National Household Sample Survey* [PNAD, acronym in Portuguese] 2013, n = 116,196, *Brazilian Household Budget Survey* 2018, n = 57,920, Continuous PNAD 2023, n = 173,676). Food security and insecurity were estimated based on the profiles of the heads of households, defined based on sex and self-reported race/skin color (white man, white woman, Mixed-race man, Mixed-race woman, Black man, Black woman). Relative risk ratios (RRR) were estimated by multinomial logistic regression models to test the trajectories of associations with food insecurity . From 2013 to 2017/2018, food security decreased in all profiles, increasing from 2018 to 2023, mainly in households headed by Black women (p-value < 0.001). The most severe forms of food insecurity remained significantly associated among households headed by Black or Brown people, being higher in those in which Black women occupied this position. Over the 10 years of the surveys, families in mild food insecurity headed by a white woman showed reduced RRR values (RRR = 1.2, 95%CI: 1.1-1.4). For those with severe food insecurity , the greatest reduction in RRR occurred in households headed by Black women (RRR = 2.1, 95%CI: 1.8-2.5). Although food insecurity decreased in Brazil in 2023, racial inequalities persist, reiterating the need for public policies to reduce the structural inequities faced by its Black population.

## Abstract (es)

El objetivo de este estudio fue evaluar la trayectoria de la inseguridad alimentaria en el país según las intersecciones de sexo y raza/color a lo largo de 2013 y 2023. Se evaluaron los microdatos de las encuestas de población (*Encuesta Nacional por Muestra de Domicilios* [PNAD, por su sigla en portugués] 2013; n= 116.196; *Encuesta de Presupuesto de Familiares* 2018; n = 57.920; PNAD Continua 2023; n = 173.676). La seguridad alimentaria y la inseguridad alimentaria se estimaron en función de los perfiles de los jefes de hogar definidos según el sexo y la raza/color autoinformados (hombre blanco, mujer blanca, hombre pardo, mujer parda, hombre negro, mujer negra). Las ratios de riesgo relativo (RRR) se estimaron mediante modelos de regresión logística multinomial para probar las trayectorias de las asociaciones con la inseguridad alimentaria. Entre 2013 y 2017/2018, la seguridad alimentaria disminuyó en todos los perfiles, seguida del aumento en el período de 2018 a 2023 principalmente entre las familias encabezadas por mujeres negras (p-valor < 0,001). Las formas más severas de inseguridad alimentaria se mantuvieron significativamente asociadas entre las familias encabezadas por personas negras o pardas, y fueron más altas en aquellas donde la mujer negra ocupaba esta posición. Durante los 10 años de las encuestas, las familias con inseguridad alimentaria leve encabezadas por una mujer blanca redujeron los valores de RRR (RRR = 1,2; IC95%: 1,1-1,4). Para aquellas con inseguridad alimentaria severa, la mayor reducción en la RRR se observó entre las familias encabezadas por una mujer negra (RRR = 2,1; IC95%: 1,8-2,5). Aunque la inseguridad alimentaria se redujo en el país en 2023, las desigualdades raciales persistieron, lo que reafirma la necesidad de políticas públicas para reducir las desigualdades estructurales que enfrenta la población negra.

## Introdução 

Em 2026, a Lei Orgânica de Segurança Alimentar (LOSAN) completará 20 anos desde a sua promulgação, marco que instituiu o Sistema de Segurança Alimentar e Nutricional com o objetivo de assegurar o direito humano à alimentação adequada (DHAA) ^1^. Esse direito pressupõe a realização da segurança alimentar e nutricional, reconhecendo que todos os cidadãos têm o direito a uma alimentação regular, suficiente e de qualidade, pautada em práticas alimentares saudáveis, culturalmente adequadas e sustentáveis nos âmbitos social, econômico e ambiental. No entanto, opressões estruturais permanecem e afetam intensamente o acesso de determinados grupos a esse direito ^2^.

Historicamente, a estrutura social e econômica brasileira foi moldada pelo racismo, um sistema de discriminação e opressão que impõe desvantagens à população negra (pretos e pardos) e aos povos indígenas, resultando em exclusão social e nos piores indicadores socioeconômicos ^3^ ^,^ ^4^. Essa desigualdade é intensificada quando se considera a perspectiva interseccional de gênero, em que mulheres negras enfrentam o “racismo genderizado”, uma articulação entre racismo e sexismo que impacta diretamente suas trajetórias sociais ^5^.

Essa perspectiva surge das reflexões de pesquisadoras negras que, diante da ausência da dimensão racial nos estudos sobre mulheres e nas pautas dos movimentos feministas, denunciaram a invisibilidade das experiências de mulheres negras. Embora o conceito de interseccionalidade tenha sido formalizado por Crenshaw (1989) ^6^ como uma abordagem voltada a compreender as consequências estruturais e dinâmicas da interação entre múltiplos eixos de subordinação, autoras brasileiras ^7^ ^,^ ^8^ ^,^ ^9^ ^,^ ^10^ já desenvolviam análises nessa direção antes dessa sistematização. Alguns anos antes, Lélia Gonzalez ^7^ ^,^ ^8^ examinou os espaços sociais ocupados por mulheres negras e articulou as inter-relações entre raça, sexo e classe, contribuindo para uma leitura que dialoga com o que posteriormente seria reconhecido como interseccionalidade.

Nesse sentido, o campo da segurança alimentar e nutricional se apresenta como um espaço fundamental para analisar tais desigualdades, uma vez que a insegurança alimentar constitui uma das principais expressões da violação do DHAA, manifestando-se desde a preocupação com a aquisição de alimentos até a restrição severa de consumo, caracterizando, em seu grau mais grave, a fome ^11^. A escritora Carolina Maria de Jesus ^12^, ao relatar em seu diário as experiências de uma mulher negra marcada pela fome, evidenciou a dimensão particular desse processo, no qual a fome é associada à cor amarela como expressão do sofrimento e da privação extrema. Dessa forma, compreendida a magnitude do problema, os debates sobre raça/cor e gênero se intensificaram, evidenciando as desigualdades na experiência da insegurança alimentar na população ^13^ ^,^ ^14^ ^,^ ^15^ ^,^ ^16^.

Esse movimento tem sido sustentado pela mensuração contínua da insegurança alimentar no país, consolidada desde 2004 com a implantação de indicadores diretos aplicados em nível domiciliar por meio da *Escala Brasileira de Insegurança Alimentar* (EBIA) ^17^ ^,^ ^18^. Baseando-se na escala aplicada regularmente em estudos populacionais, é possível identificar domicílios em situação de segurança alimentar ou em níveis distintos de insegurança alimentar, conforme as dificuldades de acesso aos alimentos.

Quando aplicada em inquéritos populacionais de grande abrangência, a exemplo das *Pesquisa Nacional por Amostra de Domicílios* (PNAD) e da *Pesquisa de Orçamentos Familiares* (POF), a EBIA também permitiu analisar a distribuição da insegurança alimentar entre diferentes grupos sociais, contribuindo para a identificação de desigualdades estruturais no acesso à alimentação, assim como os efeitos das políticas de segurança alimentar e nutricional sobre as proporções de domicílios em condição de segurança alimentar e insegurança alimentar ^19^ ^,^ ^20^. Um marco importante do monitoramento foi a PNAD 2013, que evidenciou melhora significativa nos níveis de segurança alimentar e queda expressiva na insegurança alimentar, contribuindo para a retirada do Brasil do Mapa da Fome em 2014 ^19^ ^,^ ^21^. Esse cenário foi fruto do investimento em um conjunto de ações integradas e que contaram com ampla participação da sociedade ^22^. 

Anos depois, os dados da POF 2017/2018 revelaram queda acentuada da segurança alimentar e aumento dos níveis de insegurança alimentar, reflexo de um cenário político e econômico desfavorável, e o desmonte da estrutura de políticas de segurança alimentar nutricional ^23^ ^,^ ^24^. Tais flutuações nos percentuais de segurança alimentar e insegurança alimentar ao longo dos anos estão diretamente associadas a características sociodemográficas, entre as quais, a raça/cor da pele se destaca de forma marcante ^15^ ^,^ ^25^. Em períodos de instabilidade, como nos anos posteriores a 2016 e à pandemia de COVID-19, essas desigualdades foram aprofundadas e a fome afetou com maior intensidade a população negra, especialmente as mulheres ^14^ ^,^ ^26^ ^,^ ^27^.

Considerando que a prioridade da segurança alimentar nutricional na agenda pública tem oscilado principalmente a partir de 2016, refletida nas variações dos níveis de insegurança alimentar em âmbito domiciliar, torna-se essencial monitorar e compreender como essas mudanças ocorrem diante das desigualdades raciais. Nesse contexto, o presente estudo tem como objetivo analisar a tendência da insegurança alimentar em famílias chefiadas por pessoas negras no Brasil, no período de 2013 a 2023, considerando uma abordagem interseccional entre raça/cor e gênero.

## Métodos

### Desenho de estudo e amostra

Trata-se de um estudo transversal, baseado na análise dos microdados da PNAD 2013 (116.196 domicílios) ^28^, da POF 2017/2018 (57.920 domicílios) ^29^ e da PNAD Contínua 2023 (173.676 domicílios) ^30^, todas realizadas pelo Instituto Brasileiro de Geografia e Estatística (IBGE). As edições das PNAD e PNAD Contínua abrangem diferentes tipos de domicílios, incluindo os particulares permanentes e domicílios coletivos, como hotéis, pensões, orfanatos e asilos. Por outro lado, a POF contempla exclusivamente domicílios particulares permanentes. Para assegurar a comparabilidade entre as duas pesquisas, foram consideradas, nas análises com dados da PNAD, apenas as unidades domiciliares particulares permanentes. Dessa forma, as análises realizadas garantem a equivalência entre os inquéritos quanto ao desenho amostral e à mesma metodologia utilizada para estimar a segurança alimentar e insegurança alimentar nesse tipo específico de domicílio. Mais detalhes do plano amostral dos referidos inquéritos podem ser consultados nos relatórios oficiais divulgados pelo IBGE ^9^ ^,^ ^20^ ^,^ ^31^.

### Insegurança alimentar

A EBIA foi utilizada pelos inquéritos para analisar a segurança alimentar e níveis de insegurança alimentar (leve, moderada e grave). Trata-se de escala psicométrica que avalia a percepção do entrevistado sobre o acesso aos alimentos em termos de qualidade e quantidade nos três meses anteriores à entrevista. A escala consiste em 14 perguntas dicotômicas (sim/não), sendo oito aplicadas a adultos do domicílio (com 18 anos ou mais) e as seis restantes exclusivamente a famílias com ao menos uma criança e/ou adolescentes ^32^. Os domicílios foram classificados nas categorias: segurança alimentar, insegurança alimentar leve, insegurança alimentar moderada e insegurança alimentar grave ^32^.

### Perfil das famílias

Visando a entender as relações interseccionais entre gênero e raça/cor na insegurança alimentar, foram criados perfis com as características das pessoas de referências (ou chefe do domicílio), utilizando as perguntas sobre sexo (masculino e feminino) e raça/cor autorreferidos com base na classificação do IBGE ^33^. Dado que os inquéritos utilizados não têm representatividade de grupos étnicos representados pela classificação amarela e indígena, esses foram excluídos do estudo. Dessa forma, com base no cruzamento entre sexo e raça/cor, cria-se a variável de exposição, dividida em seis perfis: homem branco, mulher branca, homem pardo, mulher parda, homem preto e mulher preta. 

### Covariáveis de estudo

Foram avaliadas as características sociodemográficas segundo os perfis dos chefes de família em cada um dos inquéritos analisados. A seleção das variáveis foi baseada em revisão da literatura, considerando aquelas identificadas como determinantes da segurança alimentar e insegurança alimentar ^11^, bem como as associadas à ocorrência de insegurança alimentar em domicílios, conforme evidenciado na revisão sistemática conduzida por Lignani et al. ^25^. Foram incluídas no estudo as variáveis disponíveis de forma compatível nos três inquéritos.

As variáveis selecionadas incluíram informações do domicílio: região geográfica (Norte, Nordeste, Sudeste, Centro-oeste e Sul), localização (urbana ou rural), número de moradores (1-2, 3-5 ou mais de 5) e renda familiar *per capita* (calculada pela razão entre a soma das rendas de todos os membros da família e o total de moradores), categorizada por tercis. Com relação ao chefe do domicílio, foram consideradas as seguintes variáveis: faixa etária e escolaridade, expressa pelos anos de estudos (0, 1-7, 8-12 e mais de 12).

### Análise de dados

As análises estatísticas consideraram a expansão dos dados para a representatividade da população brasileira. As prevalências das características sociodemográficas, de segurança alimentar e níveis de insegurança alimentar foram estimadas pelos perfis, por meio de tabelas de contingência, considerando-se o intervalo de 95% de confiança (IC95%). As diferenças entre as prevalências foram avaliadas por meio do teste qui-quadrado de Pearson. 

Para avaliar a relação entre os perfis do chefe do domicílio em relação aos diferentes níveis de insegurança alimentar em cada inquérito, modelos de regressão logística multinomial foram realizados separadamente, estimando-se as razões de risco relativo (RRR), com IC95%, adotando-se a condição de segurança alimentar e o perfil de chefe do domicílio “homem branco” como categorias de referências. 

A análise foi conduzida em duas etapas. Na primeira, foram realizados modelos bivariados não ajustados para cada perfil de chefe do domicílio e para as covariáveis de interesse. As variáveis que apresentaram associação estatisticamente significativa (valor de p < 0,05) foram incluídas na segunda etapa. Na etapa final, os modelos ajustados foram estimados, incorporando-se as covariáveis previamente identificadas como associadas, de modo a controlar potenciais fatores de confusão na relação entre o perfil do chefe de domicílio e os níveis de insegurança alimentar. As variáveis de ajuste consideradas nos modelos foram: área do domicílio, macrorregião, presença de crianças menores de dez anos, número de moradores, anos de estudos e renda familiar *per capita*.

Os efeitos marginais foram estimados para o desfecho de insegurança alimentar de acordo com os perfis do responsável pelo domicílio, após o ajuste do modelo final, separadamente para dois anos de referência (PNAD 2013 e PNAD Contínua 2023), selecionados por representarem os limites do período analisado. A variação na probabilidade prevista do desfecho foi plotada segundo os perfis do responsável pelo domicílio.

Todas as análises estatísticas foram realizadas no software Stata, versão 16.1 (https://www.stata.com), considerando-se os pesos amostrais respectivos. Este estudo utilizou bancos de dados de uso público, com informações agregadas, sem possibilidade de identificação individual dos participantes. Por essa razão, e em consonância com a *Resolução nº 510*, de 7 de abril de 2016, da Comissão Nacional de Ética em Pesquisa (CONEP), não foi necessária a submissão para aprovação em comitê de ética. 

## Resultados

A Tabela 1 apresenta as características sociodemográficas das famílias brasileiras e revela a persistência de desigualdades entre os perfis de chefia de domicílio. Em 2023, a prevalência de domicílios cujo chefe possuía maior escolaridade (12 anos ou mais de estudos) manteve-se inferior entre aqueles chefiados por homens pardos (16,2%), homens pretos (17,4%), mulheres pardas (17,4%) e mulheres pretas (17,8%), sendo mais elevada nos domicílios chefiados por homens brancos (34,1%) e mulheres brancas (34,3%).

**Tabela 1:** Características sociodemográficas das pessoas de referência e dos domicílios do Brasil segundo a raça/cor e sexo.

Características	Homem branco		Mulher branca		Homem pardo		Mulher parda		Homem preto		Mulher preta	
	%	IC95%	%	IC95%	%	IC95%	%	IC95%	%	IC95%	%	IC95%
PNAD 2013												
Idade (anos)												
Até 24	3,2	3,0-3,5	3,8	3,4-4,2	4,7	4,4-5,1	5,2	4,7-5,6	4,6	4,0-5,2	4,1	3,4-5,0
25-39	27,7	27,1-28,4	22,3	21,6-23,1	32,4	31,7-33,1	27,3	26,5-28,1	32,0	30,8-33,2	27,2	25,4-29,1
40-59	43,5	42,8-44,1	39,6	38,7-40,5	42,0	41,4-42,6	40,3	39,4-41,2	42,6	41,4-43,9	41,2	39,6-42,7
60 ou mais	25,6	24,8-26,4	34,3	33,1-35,5	20,9	20,2-21,6	27,3	26,3-28,2	20,8	19,6-22,2	27,5	25,8-29,2
Escolaridade (anos de estudos)												
Analfabeto	7,9	7,1-8,8	9,9	8,8-11,0	17,3	16,1-18,5	17,4	16,1-18,8	17,8	15,8-19,9	17,9	15,8-20,2
1-7	31,6	29,2-34,1	30,4	28,5-32,4	38,0	36,6-39,4	35,4	34,1-36,7	36,3	34,6-38,1	34,4	32,3-36,6
8-12	40,9	40,0-41,8	38,9	38,0-39,8	37,7	36,0-39,5	38,5	37,0-40,1	39,4	36,8-42,1	39,7	36,9-42,6
> 12	19,5	16,4-23,1	20,9	18,5-23,4	7,0	6,2-7,9	8,7	7,8-9,7	6,5	5,8-7,3	8,0	6,9-9,3
Domicílio												
Macrorregiões												
Norte	3,3	2,3-4,6	3,3	1,9-5,5	11,5	8,8-15,0	11,4	7,6-16,8	8,2	6,0-11,0	6,6	4,4-9,9
Nordeste	14,8	11,7-18,5	15,0	11,2-19,9	36,5	31,5-41,8	38,5	31,9-45,4	32,1	24,1-41,4	32,9	23,0-44,6
Centro-oeste	6,4	4,2-9,7	6,9	3,9-11,8	9,1	6,5-12,8	8,9	5,6-13,7	7,4	4,7-11,3	6,3	3,6-10,7
Sul	25,3	19,9-31,5	24,2	18,0-31,6	6,7	5,5-8,1	6,6	5,2-8,5	7,3	5,3-9,9	7,4	4,6-11,8
Sudeste	50,3	41,2-59,4	50,7	40,0-61,3	36,2	29,6-43,3	34,6	27,3-42,7	45,1	36,0-54,5	46,8	35,4-58,5
Área do domicílio												
Urbana	86,3	83,4-88,7	94,2	92,8-95,4	77,2	74,3-79,8	89,1	87,3-90,6	84,8	81,6-87,5	92,1	89,7-94,0
Rural	13,7	11,3-16,6	5,8	4,6-7,2	22,9	20,2-25,7	10,9	9,4-12,7	15,2	12,5-18,4	7,9	6,0-10,3
Moradores												
1-2	36,7	35,6-37,8	52,4	50,8-54,0	32,4	31,5-33,4	40,7	39,4-41,9	35,0	33,2-36,8	40,6	38,3-42,9
3-5	59,2	58,3-60,2	43,8	42,3-45,2	59,9	59,0-60,7	51,0	50,1-52,0	56,7	55,2-58,1	51,4	49,4-53,3
> 5	4,1	3,8-4,4	3,9	3,5-4,3	7,7	7,3-8,2	8,3	7,6-9,0	8,4	7,4-9,5	8,0	7,1-9,1
Presença de crianças menores de 10 anos												
Não	70,6	69,6-71,6	77,1	76,0-78,2	63,9	63,0-64,8	66,2	65,0-67,4	66,3	64,7-68,0	68,1	66,1-70,1
Sim	29,4	28,4-30,4	22,9	21,8-24,0	36,1	35,2-37,0	33,8	32,6-35,0	33,7	32,0-35,3	31,9	29,9-34,0
Renda *per capita* mensal (tercil)												
1º	20,8	18,7-23,1	22,3	20,6-24,2	40,7	38,5-43,0	43,8	41,9-45,7	37,0	34,2-39,9	42,6	39,5-45,7
2º	33,3	31,5-35,1	33,4	31,8-35,1	34,1	33,5-34,8	34,1	33,2-35,0	36,9	35,6-38,3	34,8	33,0-36,6
3º	45,9	42,1-49,7	44,2	41,1-47,5	25,1	23,1-27,3	22,1	20,4-23,9	26,1	23,9-28,4	22,6	20,6-24,9
POF 2017/2018												
Idade (anos)												
Até 24	2,7	2,4-3,1	2,8	2,4-3,3	4,4	4,0-4,9	4,4	3,9-4,8	4,0	3,3-4,8	3,6	2,8-4,5
25-39	24,2	23,3-25,2	20,9	19,8-22,0	29,6	28,7-30,5	25,7	24,7-26,7	28,9	27,1-30,7	25,0	22,9-27,2
40-59	44,1	43,0-45,2	41,0	39,7-42,2	42,6	41,6-43,6	42,2	41,1-43,4	42,9	40,9-44,9	40,3	38,1-42,6
60 ou mais	29,0	28,0-30,0	35,3	34,1-36,6	23,4	22,6-24,3	27,8	26,7-28,8	24,3	22,6-26,1	31,2	29,1-33,4
Escolaridade (anos de estudos)												
Analfabeto	5,3	4,8-5,8	7,1	6,5-7,9	11,5	10,9-12,3	12,1	11,4-12,9	12,3	11,0-13,8	14,2	12,6-16,0
1-7	36,8	35,6-38,0	32,3	31,1-33,6	39,3	38,3-40,3	36,0	35,0-37,1	37,3	35,4-39,3	35,5	33,3-37,8
8-12	39,0	37,9-40,1	35,7	34,4-37,0	39,2	38,2-40,3	37,6	36,6-38,7	40,3	38,3-42,3	35,0	32,7-37,3
> 12	18,9	17,9-20,1	24,9	23,5-26,2	9,9	9,3-10,6	14,2	13,4-15,1	10,1	8,9-11,4	15,3	13,6-17,1
Domicílio												
Macrorregiões												
Norte	6,8	6,2-7,4	7,2	6,5-7,9	19,2	18,2-20,2	18,3	17,3-19,4	14,5	12,9-16,2	12,2	10,5-14,0
Nordeste	23,7	22,4-25,0	31,2	29,7-32,8	42,8	41,5-44,2	50,7	49,3-52,2	38,9	36,7-41,2	44,1	41,5-46,8
Centro-oeste	13,2	12,3-14,2	11,2	10,2-12,3	13,5	12,6-14,3	11,3	10,4-12,2	15,9	14,2-17,6	12,4	10,7-14,4
Sul	27,2	25,6-28,8	24,7	23,0-26,5	5,6	5,0-6,3	4,9	4,2-5,6	4,0	3,3-4,9	6,7	5,5-8,1
Sudeste	29,1	27,8-30,5	25,7	24,4-27,0	18,9	18,0-19,9	14,9	14,0-15,8	26,7	24,8-28,6	24,6	22,4-26,9
Área do domicílio												
Urbana	70,5	68,9-72,0	86,0	84,7-87,2	72,4	71,1-73,7	84,4	83,2-85,6	74,5	72,4-76,6	85,4	83,1-87,5
Rural	29,5	28,0-31,1	14,0	12,8-15,3	27,6	26,3-28,9	15,6	14,4-16,8	25,5	23,4-27,6	14,6	12,5-16,9
Moradores												
1-2	38,7	37,6-39,8	50,3	48,9-51,6	34,7	33,8-35,7	39,0	38,0-40,1	35,9	33,9-37,8	39,0	36,7-41,4
3-5	50,1	49,0-51,1	39,2	37,9-40,5	47,7	46,8-48,7	42,4	41,3-43,5	46,7	44,7-48,7	43,1	40,7-45,5
> 5	11,3	10,6-12,0	10,5	9,7-11,4	17,6	16,8-18,3	18,6	17,7-19,5	17,5	16,0-19,0	17,9	16,2-19,7
Presença de crianças menores de 10 anos												
Não	72,6	71,6-73,6	77,9	76,8-79,0	65,8	64,9-66,7	67,1	66,1-68,1	66,9	65,1-68,8	68,2	66,1-70,3
Sim	27,4	26,4-28,4	22,1	21,1-23,2	34,2	33,3-35,2	32,9	31,9-33,9	33,1	31,2-34,9	31,8	29,7-33,9
Renda *per capita* mensal (tercil)												
1º	21,8	20,8-22,8	22,8	21,7-24,0	40,9	39,8-42,0	44,9	43,7-46,1	37,8	35,9-39,8	41,9	39,5-44,3
2º	32,5	31,4-33,6	33,0	31,7-34,4	33,6	32,7-34,5	32,6	31,6-33,7	36,6	34,8-38,5	34,0	31,9-36,2
3º	45,8	44,5-47,1	44,1	42,6-45,7	25,5	24,6-26,4	22,5	21,5-23,5	25,6	23,9-27,4	24,1	22,2-26,2
PNAD Contínua 2023												
Idade (anos)												
Até 24	3,4	3,1-3,7	4,7	4,4-5,1	5,3	5,0-5,7	6,5	6,2-6,9	6,3	5,6-7,1	6,8	6,1-7,6
25-39	27,4	26,6-28,2	24,8	24,1-25,5	30,9	30,2-31,6	31,4	30,8-32,0	32,2	30,7-33,6	29,7	28,5-30,9
40-59	39,6	38,8-40,3	37,8	37,1-38,5	40,8	40,1-41,5	39,0	38,4-39,6	40,2	38,8-41,6	39,5	38,2-40,7
60 ou mais	29,7	29,0-30,4	32,7	32,0-33,5	22,9	22,4-23,5	23,1	22,5-23,6	21,4	20,3-22,4	24,0	23,0-25,1
Escolaridade (anos de estudos)												
Analfabeto	3,5	3,2-3,7	3,8	3,5-4,0	7,3	6,9-7,6	6,8	6,5-7,1	7,0	6,4-7,6	6,8	6,2-7,4
1-7	21,6	21,0-22,2	21,1	20,5-21,7	28,5	27,8-29,1	26,1	25,6-26,7	25,7	24,6-27,0	25,3	24,2-26,4
8-12	40,8	40,0-41,7	40,9	40,1-41,7	47,5	46,8-48,2	49,6	49,0-50,3	49,9	48,4-51,5	50,1	48,9-51,4
> 12	34,1	33,2-35,1	34,3	33,4-35,2	16,8	16,2-17,4	17,4	16,9-18,0	17,4	16,2-18,6	17,8	16,7-18,9
Domicílio												
Macrorregiões												
Norte	3,5	3,3-3,8	3,3	3,1-3,5	12,4	12,0-12,8	11,2	10,8-11,5	8,3	7,7-9,0	6,2	5,6-6,8
Nordeste	14,0	13,5-14,6	15,5	15,0-16,0	32,4	31,7-33,1	38,6	37,9-39,3	30,9	29,6-32,3	35,3	34,0-36,8
Centro-oeste	6,9	6,5-7,2	6,3	6,0-6,6	9,8	9,4-10,2	9,1	8,7-9,4	8,4	7,7-9,1	6,6	6,1-7,2
Sul	25,6	24,9-26,3	24,9	24,3-25,6	7,7	7,3-8,1	6,7	6,3-7,0	7,6	6,9-8,3	6,9	6,3-7,5
Sudeste	50,0	49,1-50,9	50,1	49,3-50,9	37,8	36,9-38,6	34,5	33,7-35,3	44,8	43,2-46,4	45,0	43,5-46,5
Área do domicílio												
Urbana	87,7	87,2-88,2	92,9	92,6-93,2	82,4	81,9-82,9	87,8	87,4-88,2	87,5	86,8-88,2	90,8	90,0-91,5
Rural	12,3	11,9-12,8	7,1	6,8-7,4	17,6	17,1-18,1	12,2	11,8-12,6	12,5	11,8-13,2	9,2	8,5-10,0
Moradores												
1-2	51,0	50,1-51,8	51,4	50,6-52,2	47,0	46,3-47,7	40,8	40,1-41,4	49,5	48,1-51,0	42,8	41,6-44,1
3-5	47,2	46,3-48,0	46,4	45,7-47,2	49,6	48,9-50,3	54,6	53,9-55,2	46,7	45,2-48,1	52,3	51,0-53,6
> 5	1,9	1,7-2,1	2,2	1,9-2,4	3,5	3,2-3,7	4,7	4,4-5,0	3,8	3,3-4,5	4,9	4,3-5,5
Presença de crianças menores de 10 anos												
Não	87,0	86,5-87,6	84,6	84,0-85,1	84,0	83,5-84,5	77,6	77,1-78,2	85,3	84,3-86,3	79,8	78,7-80,8
Sim	13,0	12,5-13,5	15,4	14,9-16,0	16,0	15,5-16,5	22,4	21,8-22,9	14,7	13,7-15,7	20,2	19,2-21,3
Renda *per capita* mensal (tercil)												
1º	16,4	15,9-17,1	23,5	22,8-24,2	31,4	30,8-32,1	45,4	44,7-46,1	28,3	27,1-29,6	43,5	42,2-44,9
2º	27,7	27,0-28,4	30,5	29,8-31,2	35,1	34,5-35,8	33,1	32,5-33,7	37,1	35,7-38,4	34,5	33,3-35,8
3º	55,9	55,0-56,8	46,1	45,2-47,0	33,4	32,7-34,2	21,5	20,9-22,1	34,6	33,2-36,1	21,9	20,8-23,1

IC95%: intervalo de 95% de confiança; PNAD: *Pesquisa Nacional por Amostra de Domicílio*; POF: *Pesquisa de Orçamentos Familiares.*

Fonte: PNAD ^28^, POF ^29^; PNAD Contínua ^30^.

Nota: todos os valores apresentaram valor de p < 0,01 segundo o teste de qui-quadrado de Pearson.

A maioria das famílias residia em áreas urbanas, com distribuição regional se modificando entre 2013 e 2023 para a predominância no Sudeste em todos os perfis analisados, exceto os liderados por mulheres pardas, cuja maioria permaneceu no Nordeste (38,6%). Em relação à composição domiciliar, famílias chefiadas por homens e mulheres brancos passaram a compor mais o segmento de famílias com 1 a 2 moradores (51% e 51,4%, respectivamente) em 2023; aquelas chefiadas por pessoas negras continuaram na sua maioria caracterizadas pela composição de 3 a 5 residentes, principalmente famílias lideradas por mulheres pardas (54,6%). As maiores proporções de insegurança alimentar em domicílios com crianças foram verificadas na presença de mulheres pardas (22,4%) e pretas (20,2%). Quanto à renda, domicílios chefiados por uma pessoa branca, independentemente do gênero, concentravam-se majoritariamente no 3º tercil de renda.

**Figura 1:**
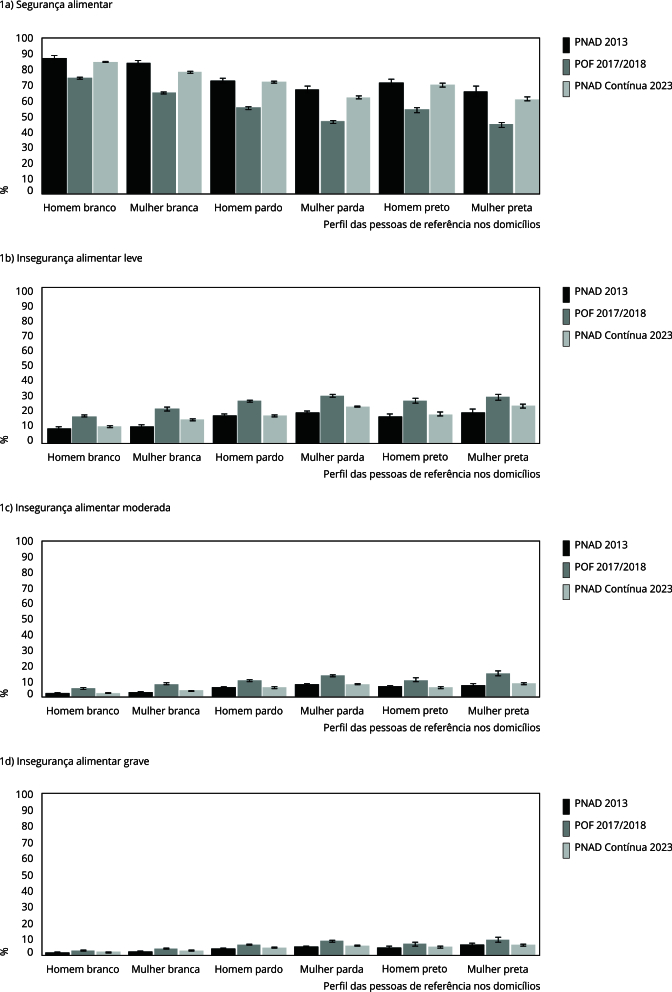
Prevalências de segurança alimentar e níveis de insegurança alimentar segundo o sexo e a raça/cor. *Pesquisa Nacional por Amostra de Domicílios* (PNAD) 2013, *Pesquisa de Orçamentos Familiares* (POF) 2017/2018, PNAD Contínua 2023, Brasil.

Ao analisar a relação entre segurança alimentar e níveis de insegurança alimentar entre os perfis, entre 2013 e 2017/2018, observou-se redução significativa da segurança alimentar em todos os grupos, especialmente em domicílios chefiados por mulheres pretas e pardas, que apresentaram trajetórias semelhantes ao longo do período. As prevalências da segurança alimentar reduziram de 66,5% (IC95%: 64,5-68,6) para 46,4% (IC95%: 45,1-47,7) e de 65,4% (IC95%: 61,9-68,7) para 44,7% (IC95%: 42,3-47,0), respectivamente, para mulheres pretas e pardas. Entre 2018 e 2023, verificou-se aumento da segurança alimentar no país, novamente com comportamento semelhante entre famílias chefiadas por mulheres pardas (61,7%; IC95%: 60,9-62,4) e pretas (60,6%; IC95%: 59,3-62,0). As prevalências de insegurança alimentar leve, moderada e grave seguiram a tendência de aumento entre 2013 e 2018 e redução em 2023, permanecendo mais elevadas entre mulheres pardas e pretas em todos os períodos analisados (Figura 1).

Fonte: PNAD ^28^, POF ^29^; PNAD Contínua ^30^. Nota: valor de p < 0,01 segundo o teste de qui-quadrado de Pearson.

**Figura 2:**
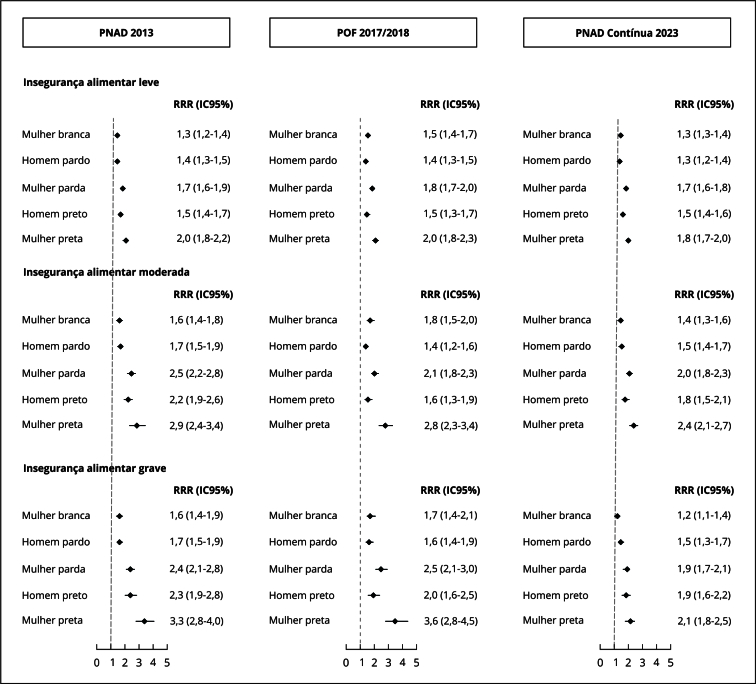
Razão de risco relativo (RRR) dos níveis de insegurança alimentar segundo o sexo e a raça/cor. *Pesquisa Nacional por Amostra de Domicílios* (PNAD) 2013, *Pesquisa de Orçamentos Familiares* (POF) 2017/2018, PNAD Contínua 2023, Brasil.

Na análise dos modelos ajustados (Figura 2) para a presença de insegurança alimentar leve, observaram-se razões de risco menores entre os domicílios chefiados por uma mulher branca ou por um homem pardo, ao longo dos anos. Apesar de as formas mais severas da insegurança alimentar (moderada e grave) permanecerem associadas a famílias chefiadas por pessoas pretas ou pardas, observou-se uma redução da RRR para insegurança alimentar grave entre famílias chefiadas por mulheres pretas, passando de RRR = 3,3 (IC95%: 2,8-4,0) em 2013 para RRR = 2,1 (IC95%: 1,8-2,5) em 2023.

Fonte: PNAD ^28^, POF ^29^; PNAD Contínua ^30^. Nota: todos os valores apresentaram valor de p < 0,01. O modelo final foi ajustado para área do domicílio, macrorregião, presença de crianças menores de 10 anos, número de moradores, anos de estudos e renda familiar *per capita*.

**Figura 3:**
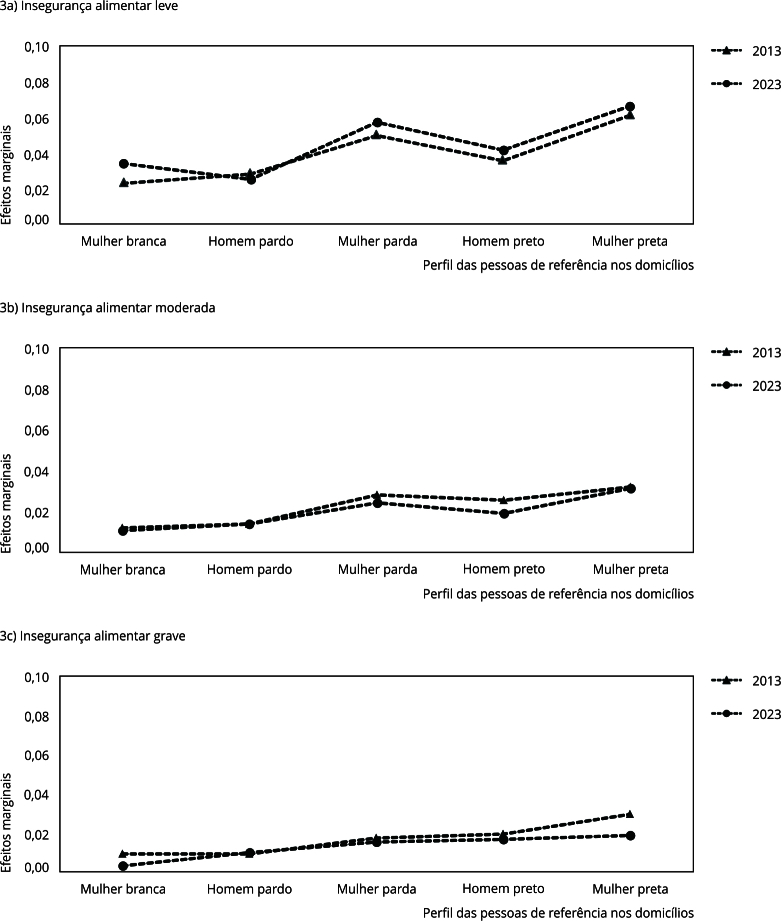
Distribuição de efeitos marginais de insegurança alimentar segundo o sexo e a raça/cor no Brasil (2013-2023).

Os efeitos marginais (Figura 3) reforçam o padrão observado nos modelos ajustados, indicando que as mudanças mais expressivas ao longo do período ocorreram principalmente na insegurança alimentar grave. Entre as mulheres pretas, a probabilidade ajustada diminuiu de 0,03 (2013) para 0,02 (2023) (valor de p < 0,001), e entre as pardas a redução foi de 0,02 para 0,01 no mesmo período (valor de p < 0,001). Para homens pretos e pardos, as probabilidades permaneceram estáveis entre 2013 e 2023, sem variações relevantes. Assim, os efeitos marginais confirmam que as principais reduções estão concentradas na insegurança alimentar grave, especialmente entre as mulheres negras.

Nota: todos os valores apresentaram valor de p < 0,05, exceto aqueles referentes a domicílios chefiados por mulheres brancas em situação de insegurança alimentar grave.

## Discussão

A última década de acompanhamento do cenário da insegurança alimentar na população brasileira permite compreender como o acesso à alimentação adequada passou por mudanças importantes, mas que ainda permanece mais desigual entre grupos mais vulneráveis, como as famílias chefiadas por mulheres pretas e pardas. Considerando que as persistentes mazelas do racismo e do sexismo continuam a impulsionar as desigualdades no Brasil ^3^, era esperado que as características sociodemográficas apresentassem diferenças significativas entre as famílias chefiadas por pessoas brancas e negras. 

A persistência dessas diferenças encontra respaldo em análises que demonstram como o racismo molda o acesso a direitos no país. Entre essas contribuições, Sueli Carneiro ^34^ explica que tais desigualdades são constituídas a partir do dispositivo de racialidade, mecanismo que organiza e hierarquiza as relações sociais ao definir a branquitude como referência de valor e humanidade, deslocando a população negra para posições sociais de menor prestígio. Quando esse cenário se articula com o sexismo, como enfatiza Carneiro ^35^, produz impactos específicos na vida das mulheres negras, que enfrentam maior precarização no trabalho, menores salários e maior concentração em ocupações de baixa remuneração, muitas delas marcadas por heranças históricas de exploração.

Esse funcionamento estrutural ajuda a compreender por que, mesmo sendo a maioria demográfica, a população negra permanece sub-representada em indicadores que refletem melhores condições de vida ^36^. Por isso, em 2022, embora pretos (10,2%) e pardos (45,3%) somassem mais da metade da população brasileira, sua participação em posições de maior proteção social e econômica continuou distante dessa proporção. Em 2021, por exemplo, a taxa de pobreza entre pessoas brancas era de 18,6%, entre pessoas pretas alcançava 34,5% e, entre pessoas pardas, 38,4% ^36^.

Embora as desigualdades persistam, observou-se um avanço importante no perfil educacional de todos os grupos entre 2013 e 2023. Esse progresso é especialmente relevante entre as famílias negras, ainda que apresentem os menores percentuais, sobretudo os homens. Estudantes homens e negros enfrentam maiores taxas de atraso, menor conclusão das etapas de ensino e maior probabilidade de evasão, compondo uma trajetória escolar marcada por práticas de racismo estrutural ^37^. Essas barreiras limitam a ascensão educacional e social e dificultam o acesso ao Ensino Superior. Nesse contexto, o marco de dez anos da Lei de Cotas é central, pois ampliou o ingresso no Ensino Superior por critérios étnico-raciais e socioeconômicos, beneficiando estudantes negros, indígenas e pessoas brancas de baixa renda ^38^. Esses avanços educacionais podem ter repercutido positivamente nas condições de vida e, consequentemente, na segurança alimentar das famílias.

Vulnerabilidades relacionadas à renda e à escolaridade, bem como ao fato de ser mulher e pertencer à raça/cor negra, também estão associadas à experiência de insegurança alimentar nos domicílios, conforme apontado na revisão sistemática conduzida por Lignani et al. ^25^. A relação entre tais características socioeconômicas e a insegurança alimentar evidenciam como a sobreposição de opressões pode levar a maiores dificuldades no acesso à educação, ao mercado de trabalho, à saúde e a outros recursos essenciais, perpetuando um ciclo de pobreza e exclusão social. Nesse contexto, as mulheres negras enfrentam múltiplas formas de discriminação e marginalização, e vivenciam restrições específicas ao alimento saudável. Os níveis de insegurança alimentar nos domicílios chefiados pelas mulheres negras têm se tornado cada vez mais evidentes nos últimos anos, especialmente com o avanço do debate interseccional aplicado ao tema ^13^ ^,^ ^14^ ^,^ ^15^ ^,^ ^16^. Esse debate refere-se à compreensão das interações entre diferentes marcadores sociais e de como suas combinações produzem formas específicas de desigualdade ^6^. 

Desse modo, os resultados encontrados refletem a perpetuação de desigualdades de forma interconectada, o que contribui para explicar as disparidades observadas nos níveis de insegurança alimentar entre os diferentes perfis familiares analisados. Evidências semelhantes também foram identificadas em estudos transversais anteriores, nos quais a prevalência de insegurança alimentar é mais elevada em domicílios chefiados por mulheres pretas e pardas ^13^ ^,^ ^14^ ^,^ ^15^ ^,^ ^16^. Além disso, a análise de diferentes perfis interseccionais, que consideram gênero, raça/cor, estado civil e presença de crianças no domicílio, evidencia a acentuada vulnerabilidade vivenciada pela população negra ^15^.

Salles-Costa et al. ^39^ destacaram que a redução da fome e da insegurança alimentar no Brasil seria especialmente impulsionada pela reestruturação de políticas públicas e ações voltadas para a redução da pobreza e das desigualdades sociais. Essa observação foi confirmada com os dados encontrados neste estudo. Nesse sentido, é importante pontuar que o 3º governo do Presidente Luís Inácio Lula da Silva adotou novamente a redução da fome como prioridade de suas ações. Dentre elas, destaca-se a implementação do “Plano Brasil sem Fome”, que engloba mais de 80 ações e programas para a redução da pobreza e da fome, destacando a reestruturação do Conselho Nacional de Segurança Alimentar e Nutricional (CONSEA) e do programa Bolsa Família com ajuste orçamentário, a reimplementação do Programa de Aquisição de Alimentos (PAA), o ajuste orçamentário do Auxílio Gás e a criação do Programa Nacional de Cozinhas Solidárias que representam um conjunto adicional de ações no enfrentamento da fome, reafirmando a sua prioridade na agenda do governo ^40^.

Tais ações realizadas nos primeiros meses de governo podem ter sido um fator determinante para a diminuição da insegurança alimentar no último trimestre de 2023. Além de todas as políticas equitativas raciais implantadas no Brasil a partir dos anos 2000, especialmente a Política Nacional de Promoção da Igualdade Racial ^41^. No Plano Brasil sem Fome, é destacado nos desafios 1.1 e 1.4 que a raça/cor é um fator determinante para a incidência da insegurança alimentar, propondo o fortalecimento dos mecanismos de identificação dos grupos mais vulneráveis à fome ^42^. Reconhecer as desigualdades raciais no desenvolvimento e na reformulação das políticas pode ter sido um elemento crucial para a redução significativa da fome nesse grupo. 

No caso do Programa Bolsa Família, embora os perfis interseccionais explorados neste estudo não constituam critérios obrigatórios para o acesso ao benefício, essas ações tendem a beneficiar prioritariamente as populações em situação de maior vulnerabilidade. O programa foi retomado com reajuste de valores e passou a considerar o tamanho e as características das famílias brasileiras, incluindo uma nova cesta de benefícios e a valorização da primeira infância ^40^. Em anos anteriores, esse benefício já foi apontado como um fator de proteção contra a insegurança alimentar grave na área rural, além de ter se mostrado protetor para os níveis mais severos da insegurança alimentar na área urbana da Região Nordeste, por exemplo ^43^. De forma semelhante, outras análises indicam que o rendimento do Programa Bolsa Família tem participação significativa na composição da renda de famílias em situação de insegurança alimentar, especialmente entre as mais pobres e com chefia feminina, tanto em áreas urbanas quanto rurais ^44^. Ainda no que diz respeito ao fator renda, o aumento do salário mínimo para R$ 1.320,00 representa um elemento que pode ter contribuído para ampliar o poder de compra das famílias, favorecendo a aquisição de alimentos em quantidade e qualidade adequadas ^45^.

Embora os resultados deste estudo mostrem que a renda familiar *per capita* tenha piorado em 2023 em comparação a 2017-2018, é importante considerar que, entre esses períodos, ocorreu a pandemia de COVID-19, durante a qual a renda das famílias foi fortemente impactada, sobretudo nos domicílios chefiados por mulheres negras, o que agravou ainda mais a insegurança alimentar nesses contextos ^26^ ^,^ ^27^. O suplemento *Insegurança Alimentar e Desigualdades de Raça/Cor da Pele e Gênero* ^26^ mostra que, em 2021, domicílios chefiados por mulheres negras com rendimento igual ou superior a 1/2 salário mínimo *per capita* apresentaram 19,8% de insegurança alimentar grave e moderada, já entre os domicílios chefiados por homens brancos na mesma faixa de renda, esse porcentual foi de 8,4%. Ou seja, é possível que os resultados apresentados neste estudo reflitam ainda os efeitos persistentes da crise econômica e social que foi agravada pela pandemia, além do cenário político-econômico mundial mais recente, que tem impactado na economia de diversos países como o Brasil.

Outro destaque importante foi o relançamento do PAA, que havia sido desmontado em anos anteriores e foi retomado em novo formato, estabelecendo prioridade para os agricultores familiares incluídos no Cadastro Único (CadÚnico), bem como para os agricultores pertencentes aos povos indígenas, comunidades quilombolas e tradicionais, assentados da reforma agrária, negros, mulheres e juventude rural ^46^. Silva et al. ^47^ destacam que, para garantir a eficácia e a sustentabilidade do PAA, é essencial consolidá-lo como uma política de Estado, assegurando financiamento contínuo e estruturação adequada. Essa consolidação é fundamental para o enfrentamento das desigualdades estruturais que comprometem a segurança alimentar de grupos historicamente vulnerabilizados. Para isso, ações interministeriais são essenciais, pois fortalecem a articulação entre diferentes políticas voltadas à promoção da equidade.

Nesse sentido, de forma inovadora, foi criado, em 2023, o Ministério da Igualdade Racial, que pode ter desempenhado um papel relevante na articulação das ações, uma vez que passou a integrar a Câmara Interministerial de Segurança Alimentar e Nutricional ^48^. Os “Pacotes pela Igualdade Racial” que reúnem diversas iniciativas e ações voltadas à promoção da equidade racial, também podem ter atuado como catalisadores na redução das desigualdades raciais no campo da segurança alimentar e nutricional ^49^. Destaca-se, ainda, o Acordo de Cooperação Técnica (ACT) firmado entre o Ministério da Igualdade Racial e o Ministério do Desenvolvimento e Assistência Social, Família e Combate à Fome, que reafirma o compromisso conjunto com a construção de uma agenda de enfrentamento da fome, da insegurança alimentar e da pobreza, por meio da qualificação dos serviços e equipamentos da assistência social ^49^.

Os achados deste artigo evidenciam a importância das políticas públicas para a recuperação da segurança alimentar nos domicílios brasileiros, especialmente quando voltadas ao enfrentamento das desigualdades sociais estruturais que ainda persistem na realidade do país. Embora os resultados indiquem melhora no cenário recente, as mudanças apresentadas neste estudo não se distribuíram de modo equânime. Domicílios chefiados por mulheres pretas e pardas seguem apresentando níveis mais elevados de insegurança alimentar, o que pode estar relacionado ao curto intervalo entre a retomada das políticas e a realização do inquérito, limitando a observação de seus efeitos cumulativos. Ainda assim, essa persistência também aponta para condicionantes estruturais que modulam o alcance e o ritmo dos impactos das ações públicas.

Bento ^50^ descreve o “pacto narcísico da branquitude” como um dispositivo que preserva posições de poder e privilégio entre pessoas brancas, restringindo mudanças estruturais mais amplas. De forma convergente, Werneck ^51^ evidencia como o racismo institucional se expressa na produção de resultados desiguais mesmo em políticas concebidas sob princípios universalistas. Esses elementos ajudam a compreender por que, apesar de avanços gerais, a redução insegurança alimentar em domicílios chefiados por mulheres negras ocorre de forma mais lenta, refletindo a interseção entre raça e gênero na experiência da insegurança alimentar no país ^51^.

Nesse sentido, a incorporação de uma perspectiva interseccional nas políticas públicas revela-se fundamental para que a população vulnerável às interações entre diferentes sistemas de opressão, como o racismo e o sexismo, consiga acessá-las de forma mais eficaz. Essa abordagem contribui para a redução mais expressiva da insegurança alimentar nesse grupo, conforme demonstrado nos resultados. No contexto recente das políticas públicas brasileiras, o III Plano Nacional de Segurança Alimentar e Nutricional (2025-2027) explicita o enfrentamento ao racismo e a promoção da equidade de gênero como eixos estruturantes, prevendo estratégias intersetoriais de combate à fome voltadas à população negra, quilombola, indígena e a povos e comunidades tradicionais, como a agenda intersetorial com foco em raça e gênero e o Programa Aquilomba Brasil ^52^. Destaca-se, no entanto, que tais ações precisam ser contínuas e fortalecidas, uma vez que esses grupos permanecem entre os mais expostos à insegurança alimentar, a fim de evitar novos cenários de crise e fome, sobretudo em domicílios chefiados por pessoas negras e por mulheres.

Como limitação, destaca-se a ausência de análise do período da pandemia de COVID-19. Embora existam dois inquéritos conduzidos durante esse período (I e II VIGISAN - Inquérito Nacional da Insegurança Alimentar), realizados pela Rede PENSSAN (Rede Brasileira de Pesquisa em Soberania e Segurança Alimentar e Nutricional), diferenças metodológicas na amostragem impedem a comparabilidade com os demais inquéritos utilizados.

## Considerações finais

Em síntese, os resultados indicam que 2023 marcou um ponto de virada na trajetória de aumento da insegurança alimentar no Brasil, com a recuperação da segurança alimentar nos domicílios e os avanços nos indicadores sociodemográficos em todos os perfis de chefes de domicílio, especialmente nos chefiados por pessoas negras que, embora apresentem melhora, ainda enfrentam profundas desigualdades em relação à população branca. Contudo, é importante destacar que uma parcela significativa das famílias brasileiras permaneceu em situação de insegurança alimentar em 2023, evidenciando que o enfrentamento da fome ainda demanda esforços contínuos.

A análise sob a perspectiva racial evidencia que a retomada e a criação de políticas públicas de combate à fome e à pobreza têm impacto mais expressivo nos lares em maior vulnerabilidade, reforçando a necessidade de manter e aprimorar essas ações a fim de que o avanço na redução da insegurança alimentar entre grupos racializados seja mais acelerado e sustentável, garantindo equidade e justiça social.

Esse avanço repercutiu na recente indicação da saída do Brasil do Mapa da Fome da Organização das Nações Unidas para a Alimentação e a Agricultura/Organização Mundial da Saúde (FAO/WHO), segundo o relatório *The State of Food Security and Nutrition in the World 2025* ^53^. É a 2ª vez que temos essa conquista, reafirmando que, quando a fome é tratada como prioridade de governo, é possível alcançar mudanças significativas no acesso à alimentação em quantidade e qualidade. Apesar dos progressos, é imprescindível manter o monitoramento contínuo, sobretudo à luz dos novos dados nacionais divulgados em outubro de 2025, assegurando que o combate à fome esteja sempre articulado ao enfrentamento das desigualdades raciais históricas que caracterizam a insegurança alimentar no país.

## Data Availability

As fontes de informação utilizadas no estudo estão indicadas no corpo do artigo.
